# Kesterite Solar Cells: Insights into Current Strategies and Challenges

**DOI:** 10.1002/advs.202004313

**Published:** 2021-03-03

**Authors:** Mingrui He, Chang Yan, Jianjun Li, Mahesh P. Suryawanshi, Jinhyeok Kim, Martin A. Green, Xiaojing Hao

**Affiliations:** ^1^ School of Photovoltaic and Renewable Energy Engineering University of New South Wales New South Wales Sydney NSW 2052 Australia; ^2^ Department of Materials Science and Engineering Chonnam National University Gwangju 61186 Republic of Korea

**Keywords:** alkali doping, bandgap engineering, kesterite, thermal treatment, thin film solar cell

## Abstract

Earth‐abundant and environmentally benign kesterite Cu_2_ZnSn(S,Se)_4_ (CZTSSe) is a promising alternative to its cousin chalcopyrite Cu(In,Ga)(S,Se)_2_ (CIGS) for photovoltaic applications. However, the power conversion efficiency of CZTSSe solar cells has been stagnant at 12.6% for years, still far lower than that of CIGS (23.35%). In this report, insights into the latest cutting‐edge strategies for further advance in the performance of kesterite solar cells is provided, particularly focusing on the postdeposition thermal treatment (for bare absorber, heterojunction, and completed device), alkali doping, and bandgap grading by engineering graded cation and/or anion alloying. These strategies, which have led to the step‐change improvements in the power conversion efficiency of the counterpart CIGS solar cells, are also the most promising ones to achieve further efficiency breakthroughs for kesterite solar cells. Herein, the recent advances in kesterite solar cells along these pathways are reviewed, and more importantly, a comprehensive understanding of the underlying mechanisms is provided, and promising directions for the ongoing development of kesterite solar cells are proposed.

## Graphical abstract

Kesterite solar cell has emerged as one of the most promising eco‐friendly and low‐cost thin film photovoltaic materials. This progress report presents not only overall grasp but also detailed insights and prospective directions of the cutting‐edge strategies in this field, particularly focusing on the postdeposition/heterojunction/device thermal treatment, alkali doping, and bandgap grading, which are promising to lead to step‐change improvements.

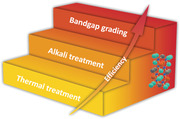

## 1 Introduction

Thin film photovoltaic (PV) technologies offer more versatility than silicon (Si) owing to their compelling features of light‐weight and compatibility to both flexible and rigid substrates, tuneability of light spectrum response for single‐junction and tandem devices, and compatibility to both opaque and semitransparent architectures.^[^ ^1^, ^2^, ^3^, ^4^ ^]^ They are an important branch of photovoltaics in realizing cost‐effective, durable and compatible products for future multiple PV markets, such as build‐integrated PV, vehicle integrated PV, semitransparent PV, flexible PV as well as weak light PV, etc.^[^ ^5^, ^6^, ^7^, ^8^ ^]^ In this area, chalcogenide CdTe and Cu(In,Ga)(S,Se)_2_ (CIGS) materials‐based solar cells have demonstrated beyond 20% efficiency with long‐term stability and have been commercialized for more than a decade.^[^ ^9^, ^10^ ^]^ However, their market share has stagnated due to, not only the rapid fall in the cost of Si‐based PV, but also the use of rare (In, Ga) and toxic (Cd) constituents.^[^ ^11^, ^12^ ^]^ These either limited the cost reduction or raise environmental concerns, thus undermining the competitiveness of chalcogenide PV compared to Si PV. Hence, sustainable terawatt level deployment of photovoltaics will eventually require the design and development of compounds based on critical raw materials (CRM)‐free and environmentally benign materials and fabrication processes.^[^ ^13^ ^]^

Kesterite Cu_2_ZnSn(S,Se)_4_ (CZTSSe) materials, comprising only non/low‐toxic and earth‐abundant constituents, have drawn the most attention and possess the highest performance (12.6%)^[^ ^14^, ^15^ ^]^ among all emerging inorganic thin film PV candidates including Se, SnS, Cu*_x_*S, FeS_2_, Cu_2_SnS_3_, Cu_2_O, and Sb_2_(S,Se)_3_.^[^ ^16^, ^17^, ^18^, ^19^, ^20^, ^21^, ^22^ ^]^ Kesterite belongs to the adamantine family of chalcogenides, as its crystal structure is derived from that of CIGS by replacing two group III cations in CIGS with one group II (Zn) and one group IV (Sn) cations.^[^ ^23^ ^]^ This cross‐substitution cation mutation brings large flexibility in element selection, thus enabling the incorporation of earth‐abundant constituents. Owing to the similar lattice and energy band structure with CIGS, kesterite‐based materials inherit the merits of the high absorption coefficient (over 10^4^ cm^−1^), tunable bandgap energy between an optimal region from 1.0 to 1.5 eV, intrinsic p‐type conductivity within a suitable range for solar cells, and the three‐dimensional symmetry of carrier transport.^[^ ^23^ ^]^ Given these similarities, kesterite has been introduced with an identical device architecture to that of CIGS (i.e., substrate configuration with Mo as back contact and CdS as electron buffer), facilitating the initial progress of this low‐cost PV technology.^[^ ^14^, ^15^ ^]^ Kesterite solar cells have reached 12.6% efficiency, which is, however, still far below its counterpart CIGS, as well as commercially competitive levels (>15%). The major barrier currently limiting the performance of kesterite solar cells is the large open‐circuit voltage (*V*_oc_)‐SQ deficit (defined by *V*_oc_ voltage difference respect to the Shockley–Queisser limit), ≈344 mV for champion CZTSSe versus ≈104 mV for champion CIGS.^[^ ^9^, ^15^, ^24^ ^]^ It is generally attributed to the large number of detrimental electronic defects within the kesterite absorber that lead to the distinct band‐ or potential‐fluctuation, short minority carrier lifetime, and associated severe bulk as well as interface recombination.^[^ ^25^, ^26^, ^27^, ^28^, ^29^, ^30^, ^31^, ^32^, ^33^, ^34^, ^35^ ^]^

These defect‐related concerns are emphasized in the flexible quaternary kesterite structure.^[^ ^29^ ^]^ The comparable ionic radii of Cu^+^ and Zn^2+^ as well as multinary oxidation states of Sn (Sn^2+^ and Sn^4+^) induce much more complicated intrinsic defect and defect cluster systems, as well as serious competitive secondary phases.^[^ ^29^, ^36^ ^]^ A variety of detrimental defects and charge compensated defect clusters may co‐exist in the kesterite lattice due to their relatively low formation energies compared to those of the electronically benign shallow defects, as suggested by advanced first‐principle calculations.^[^ ^28^, ^29^ ^]^ To suppress the formation of detrimental deep defects (Cu_Sn_, V_Sn_, Sn_Zn_) and the metal‐like Cu_2−_ *_x_*S secondary phase, an appropriate Cu‐poor (Cu/Sn<2, Cu/Zn+Sn<0.9) and Zn‐rich (Zn/Sn>1) overall composition has become the prerequisite for high performance kesterite devices.^[^ ^14^, ^15^, ^37^, ^38^ ^]^ Extensive research on engineering the chemical composition together with modifying the growth process have led to beyond 10% efficient devices in the past decade, and new strategies that will bring breakthrough improvement are urgently needed. Recent research strategies have shifted to the introduction of additional thermal treatments and extrinsic doping/alloying.^[^ ^37^, ^39^, ^40^, ^41^, ^42^, ^43^, ^44^, ^45^, ^46^, ^47^, ^48^, ^49^, ^50^, ^51^, ^52^, ^53^, ^54^, ^55^, ^56^ ^]^ These can be classified as postdeposition annealing, heterojunction or device annealing for alleviating the metastable defects and defect clusters, alkali doping for passivating bulk and interface defects, cation alloying and elemental depth profile engineering for bandgap grading.^[^ ^37^, ^39^, ^40^, ^41^, ^42^, ^43^, ^44^, ^45^, ^46^, ^47^, ^48^, ^49^, ^50^, ^51^, ^52^, ^53^, ^54^, ^55^, ^56^ ^]^ These strategies were introduced from the successful experience and high similarity of both material and device structure between kesterite CZTSSe and chalcopyrite CIGS solar cells.^[^ ^57^, ^58^, ^59^ ^]^ However, the different chemistry of these two kinds of solar cells make these strategies successful in CIGS, but might not be directly applicable to CZTSSe. Though considerable efforts have been made by employing these strategies and positive feedbacks have been obtained, the expected step‐change improvement has not yet been realized in state‐of‐the‐art kesterite solar cells. Herein, we focus on current strategies for high‐performance kesterite solar cells, including postdeposition/heterojunction/device annealing, alkali doping/alloying, and band grading engineering. In this review, we summarize the past efforts and achievements of these strategies, and more importantly, provide insights and potential directions to exploit the full potential of kesterite solar cells.

## 2 Postdeposition and Heterojunction/Device Annealing

Postannealing and related heat‐light soaking treatments have been shown effective in passivating the detrimental metastable defects and improving the performance of CIGS solar cells.^[^ ^57^, ^60^, ^61^, ^62^ ^]^ In CIGS, the metastable states like V_Cu_+V_Se_ usually have relatively large formation energies and their formation is largely attributed to the insufficient defect relaxation or anion supply during the quenching process.^[^ ^63^, ^64^ ^]^ Therefore, additional treatment with or without oxygen could effectively remove these metastable states. With the reasoning that the detrimental metastable states in the kesterite absorber could be removed in a similar way, the postannealing treatment was introduced for kesterite. This has been further applied to heterojunction and device heat treatment to facilitate the interface element interdiffusion and associated interface defect passivation, respectively, as listed in **Table** **1**. Generally, postannealing treatment can be classified into two categories: i) postdeposition (absorber) annealing and ii) heterojunction and device annealing. Herein, we reviewed the progress of these annealing treatments on kesterite solar cells and discussed the underlying mechanisms, as well as the potential and limitations of these treatments.

**Table 1:** Summary of high‐performance kesterite solar cells with thermal treatment at different fabrication stages (N/A: not available)

Absorber materials	Annealing type	Temperature [˚C]	Efficiency [%]	*V*_oc_ [mV]	*J*_sc_ [mA cm^−2^]	FF [%]	Absolute efficiency increase [%]	Ref.
CZTSSe	Absorber	300	9.7	446	32.7	67.0	5.2	^[^ ^168^ ^]^
CZTSSe	Absorber	290	10.2	502	28.8	70.5	1.4	^[^ ^49^ ^]^
CZTSSe	Absorber	300–400	11.0	480	34.0	68.0	N/A	^[^ ^50^ ^]^
CZTS	Absorber	290	7.7	658	16.7	69.5	1.4	^[^ ^49^ ^]^
CZTS	Heterojunction	270	11.0	730	21.7	69.2	3.1	^[^ ^37^ ^]^
CZTS	Heterojunction	330	9.4	700	21.3	63.0	1.8	^[^ ^77^ ^]^
CZTSSe	Device	400	11.8	485	37.5	64.9	N/A	^[^ ^166^ ^]^
CZTSSe	Device	270	8.0	424	31.6	59.6	3.6	^[^ ^91^ ^]^
CZTSe	Device	200	8.3	412	31.3	64.0	6.1	^[^ ^66^ ^]^
CZTS	Device	150	9.1	759	N/A	N/A	1	^[^ ^90^ ^]^
CZTSe‐Ge	Device	250	11.8	463	38.3	66.3	N/A	^[^ ^42^ ^]^
CZTS‐Cd	Device	300	12.6	640	27.8	71.0	5.9	^[^ ^94^ ^]^

### 2.1 Postdeposition (Absorber) Annealing

Postdeposition annealing (i.e., annealing of bare absorber) has recently been proven to be an effective approach for improving the optoelectronic quality of the kesterite absorber.^[^ ^49^, ^50^, ^65^, ^66^, ^67^, ^68^, ^69^, ^70^, ^71^, ^72^, ^73^ ^]^ In the following discussion, the annealing process performed in the air is referred to as air‐annealing. These postdeposition annealing methods on kesterite can primarily lead to the change of ordering degree of the absorber, and also the change of the bandgap and consequently *V*_oc_ and  short‐circuit current (*J*_sc_).^[^ ^68^, ^69^, ^74^ ^]^ Other positive effects such as the enhanced Na diffusion, modification of absorber surface composition as well as grain boundary passivation can be observed as well.^[^ ^49^, ^50^, ^66^, ^70^ ^]^

Theoretical calculations have revealed that the kesterite structure is prone to having cation disordering related defects such as Cu_Zn_, Cu_Sn_, Zn_Cu_, Zn_Sn_, Sn_Cu_, and Sn_Zn_, and related thermodynamically stable defect clusters.^[^ ^29^ ^]^ Among these point defects and defect clusters, the formation energy of the antisite defect cluster Cu_Zn_+Zn_Cu_ demonstrates the lowest value, indicating a large population of these disordering clusters may commonly exist in the kesterite lattice, leading to potential and/or bandgap fluctuations and thereby contributing to large *V*_oc_ deficit.^[^ ^29^ ^]^ This indicates that the typical phenomenon of Cu‐Zn order‐disorder in kesterite may be associated with bandgap variations.^[^ ^69^, ^74^ ^]^ Based on the first principle calculation, other defect clusters such as 2Cu_Zn_+Sn_Zn_ (also with very low formation energy), and Zn_Sn_+Sn_Zn_ could cause more severe band tailing if the number of these defect clusters is large.^[^ ^25^, ^29^, ^30^, ^31^ ^]^ The quantity of point defects and defect clusters is mainly determined by the formation environment, including the local chemical composition, the oxidation states, as well as the formation energy and the quenching process.^[^ ^25^, ^29^, ^31^, ^69^ ^]^ When the local coordination at high temperature largely deviates from the ordered kesterite structure, due to the limited interdiffusion within the crystal grain, large numbers of disordering defects may form in the kesterite lattice.^[^ ^67^, ^69^ ^]^ Under the conditions that the global composition is appropriate (generally Cu‐poor and Zn‐rich) and the quenching process is slow enough, the sufficient interdiffusion and lattice relaxation will significantly reduce the defects and defect clusters with high formation energy, but the defects and defect clusters with low formation energy still remain at room temperature.^[^ ^29^, ^69^, ^74^, ^75^ ^]^ Postdeposition annealing thus comes into play by introducing additional low temperature heating and quenching processes, which may lead to additional entropy variation for defect generation and relaxation processes. This could effectively change the type and quantity of intrinsic defects and defect clusters of a kesterite absorber.

**Figure 1:**
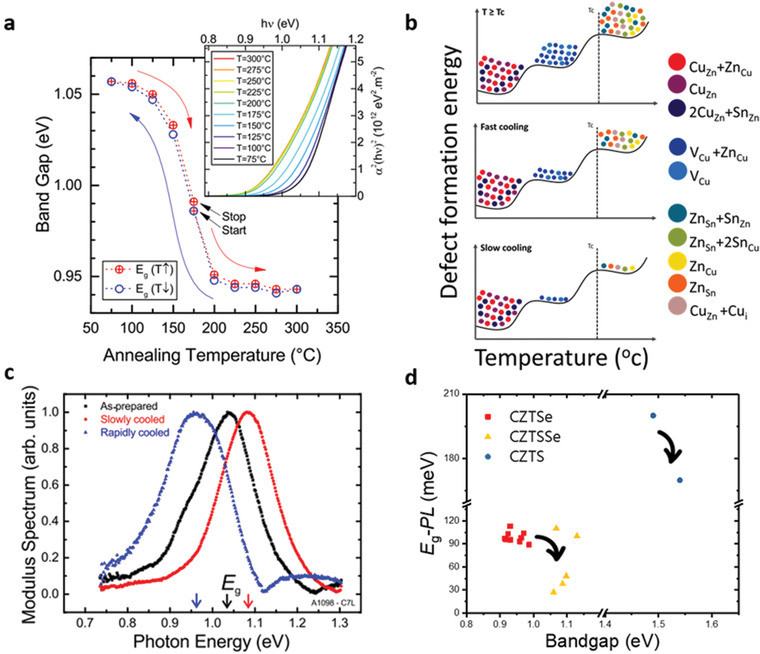
a) CZTSe bandgap versus the annealing temperature during the annealing/quenching condition. Inset: Tauc's plots for direct bandgap for lowering temperature. Reproduced with permission.^[^ ^73^ ^]^ Copyright 2014, AIP Publishing LLC. b) Schema of defect and defect cluster states for the absorber annealed above critical temperature, with fast cooling, and with slow cooling rate, respectively. The defect formation energy selected from ref. ^[^ ^29^ ^]^. c) CZTSSe bandgap shifts induced by slow and rapid cooling rate. Reproduced with permission.^[^ ^69^ ^]^ Copyright 2014, AIP Publishing LLC. d) Diagram of *E*_g_–PL versus bandgap estimated from EQE, showing degree of band tail as function of ordering–disordering. The data are selected from refs. ^[^ ^41^, ^68^, ^166^, ^167^ ^]^.

Experimentally, it has been demonstrated that the ordering degree is mostly determined by the postannealing temperature and cooling rate.^[^ ^69^, ^72^, ^73^ ^]^ A key indicator of the order parameter is marked by *S* and is derived from the Vineyard model (**Figure** **1**a),^[^ ^76^ ^]^ which gives the degree of ordering in a range of 0 (complete disordered state) to 1 (perfect ordered state). According to current research results, there are different critical temperatures (*T*_c_) for CZTSSe (195 °C), CZTS (260 °C), and CZTSe (200 °C) that can lead to a transition between ordered and disordered structures.^[^ ^69^, ^72^, ^73^ ^]^ As the significant reduction of disordering occurs by minimizing defects and defect clusters, the ordered one shows a slightly higher bandgap compared to that of the disordered one (Figure 1a).^[^ ^73^ ^]^ The cooling rate is also shown to influence the order–disorder states strongly.^[^ ^69^ ^]^ As shown in Figure 1c, a slow cooling rate (3 K h^−1^) yields kesterite films with higher bandgaps, whereas a fast cooling rate lowers the bandgap significantly. This is because the slow cooling rate enables the relaxation of defects and clusters with high formation energy, while the fast cooling rate suppresses this relaxation. The schematic diagram in Figure 1b demonstrates the defect relaxation processes, which suggest that an optimal cooling rate after the sulfurization/selenization process is critical to the formation of an ordered kesterite system. Though the number of detrimental defects in kesterite could be suppressed to some extent by controlling the postannealing process, it seems that this treatment does not work well for defects and defect clusters with low formation energy such as 2Cu_Zn_+Sn_Zn_ and Cu_Zn_+Zn_Cu_ clusters (Figure 1b).^[^ ^65^, ^66^ ^]^ This is evidenced by the fact that the photoluminescence (PL) red shift of ordered and disordered CZTSe films is not apparently influenced by the reduced defect concentration, suggesting that the band tailing states still persist (Figure 1d).^[^ ^68^, ^73^ ^]^ Interestingly, besides ordering and disordering transition, it is observed that postdeposition annealing with a fast cooling rate usually leads to increased p‐type doping density and associated higher fill factor, while that with a slow cooling rate usually leads to lower doping density and lower fill factor.^[^ ^68^ ^]^ This indicates the postdeposition annealing combined with fast cooling rate may create more cation disordering defects and more apparent acceptor‐like defects (Cu_Zn_ or Zn_Sn_) in the kesterite matrix, leading to more disorder and higher apparent p‐type doping density. Contrastingly, the postannealing combined with a slow cooling rate provides a sufficient defect relaxation process, which reduces the metastable cation disordering defects and defect clusters with high formation energy. This facilitates the formation of more stable Cu_Zn_+Zn_Cu_ clusters, leading to an increased bandgap and a reduced p‐type doping density (Cu_Zn_ defects are compensated by Zn_Cu_ defects) (Figure 1b).^[^ ^29^, ^30^, ^74^ ^]^ The band tailing states caused by 2Cu_Zn_+Sn_Zn_ clusters are stubborn and seem unchanged due to their relatively low formation energy, which explains why the detrimental bandgap fluctuation cannot be alleviated by ordering treatment.^[^ ^29^ ^]^

In addition to reducing the number of disordering states, postannealing also enables more Na diffusion from soda‐lime glass (SLG) substrate to absorber, which may benefit the performance of kesterite solar cell.^[^ ^70^ ^]^ This benefit seems to be saturated to some degree as long annealing duration, enabling more Na diffusion does not give rise to higher performance as mentioned above.^[^ ^69^ ^]^

**Figure 2:**
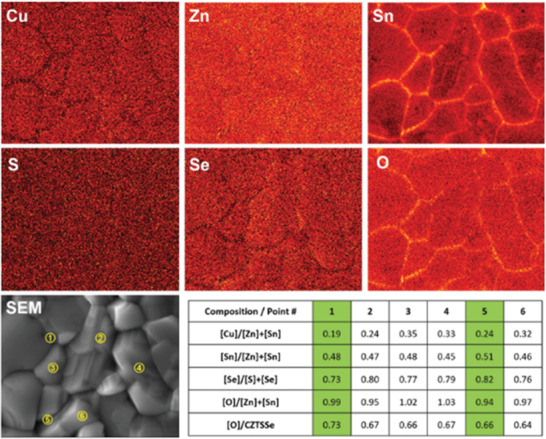
Elemental mapping for CZTSSe thin film after annealing in the air, SnO*_x_* can be found within grain boundaries. Reproduced with permission.^[^ ^50^ ^]^ Copyright 2015, Wiley‐VCH.

Compared to other postannealing treatments, the key feature of air‐annealing is that the annealing process is performed in an air atmosphere, where oxygen is ready to react with the kesterite film at surface and grain boundaries.^[^ ^49^, ^50^ ^]^ Most of the oxides formed on the surface of the absorber after air treatment can be removed by NH_4_OH etching. However, the SnO*_x_* phase can remain at grain boundaries.^[^ ^50^ ^]^ Unlike other oxides, SnO*_x_* is found to play a passivation role at grain boundaries, as shown in **Figure** **2**. Another interesting finding of air‐annealing is that oxygen could partially substitute Se sites in the kesterite lattice as a result of the formation of a thin intergranular oxide layer.^[^ ^49^ ^]^ The results of density functional theory (DFT) show that the partial substitution of Se with O can lower the valence band maximum (VBM), resulting in an increased bandgap.^[^ ^49^ ^]^ For air annealed samples, due to the formation of several oxide phases, subsequent chemical etching is necessary to remove the detrimental oxides prior to the deposition of the buffer layer.^[^ ^50^ ^]^

The recent progress in post‐ and air‐annealing treatments and associated process conditions are systematically summarized in **Table** **2**. Combined with surface etching treatment, pronounced efficiency improvements compared to the references are achieved in these cases. The enhancements achieved by introducing treatments could be mainly attributed to modified ion coordination near the surface and the corresponding relaxation of detrimental defects and defect clusters with high formation energy. Other effects such as enhanced p‐type doping caused by the reduced charge compensated donor like defects as well as the passivation of surface and grain boundaries by introducing oxides like SnO*_x_* can also be observed after treatments. Nevertheless, postdeposition annealing could not suppress the detrimental band tailing defect clusters with low formation energy, particularly the 2Cu_Zn_+Sn_Zn_ clusters. The prevalent bandgap fluctuation and associated large *V*_oc_‐deficit seem unlikely to be effectively solved by such treatments. Thus, the post‐ and air‐annealing treatment could be an effective strategy to boost the device performance when the baseline efficiency is relatively low (i.e., the absorbers suffer from detrimental defects and defect clusters with high formation energy).

**Table 2:** Summary of kesterite absorbers with post‐ and air‐annealing treatment. The detailed experimental conditions and etching solution are presented in this table. *: the etching process is prior to treatment

	Absorber	Optimized process conditions	Etching solution	Ref. efficiency [%]	Champion efficiency [%]	*V*_oc_ [mV]	*J*_sc_ [mA cm^−2^]	FF [%]	Ref.
Postannealing	CZTSSe	200 °C for 30 min with N_2_	HCl and KCN*	5.5	8.8	414	34.0	62.7	^[^ ^169^ ^]^
	CZTSe	200 °C for 30 min with Ar	KMnO_4_ + H_2_SO_4_ and Na_2_S*	1.0	5.8	344	30.2	55.6	^[^ ^66^ ^]^
	CZTSSe	300 °C for 60 min with N_2_	(NH_4_)_2_S	Unknown	7.1	466	27.4	64.7	^[^ ^70^ ^]^
Air‐annealing	CZTSSe	300 °C for 10 min	NH_4_OH	4.5	9.7	446	32.7	67.0	^[^ ^168^ ^]^
	CZTSSe	300–400 °C for several min	NH_4_OH	Unknown	11.0	480	34.0	68.0	^[^ ^50^ ^]^
	CZTSSe	290 °C for 10 min	Unknown	8.8	10.2	502	28.8	70.5	^[^ ^49^ ^]^
	CZTS	290 °C for 10 min	Unknown	6.3	7.7	658	16.7	69.5	^[^ ^49^ ^]^

### 2.2 Heterojunction and Device Annealing

Another widely adopted additional heat treatment strategy is the heterojunction/device annealing, i.e., heat treatment is applied after the deposition of the buffer layer/the completion of the device.^[^ ^37^, ^39^, ^77^, ^78^, ^79^ ^]^ Besides the advantages of improving absorber quality as discussed above, the heterojunction heat treatment (HJT) offers additional benefit by improving heterojunction interface qualities. The heterojunction interface is where the most nonradiative recombination occurs, thus governing the device performance, especially for sulfide kesterite.^[^ ^33^, ^34^, ^35^ ^]^ HJT induced interface element interdiffusion is beneficial to modify the conduction band alignment and thus reduce nonradiative recombination at the junction interface.^[^ ^37^, ^39^, ^65^, ^66^, ^77^, ^78^, ^79^ ^]^

**Figure 3:**
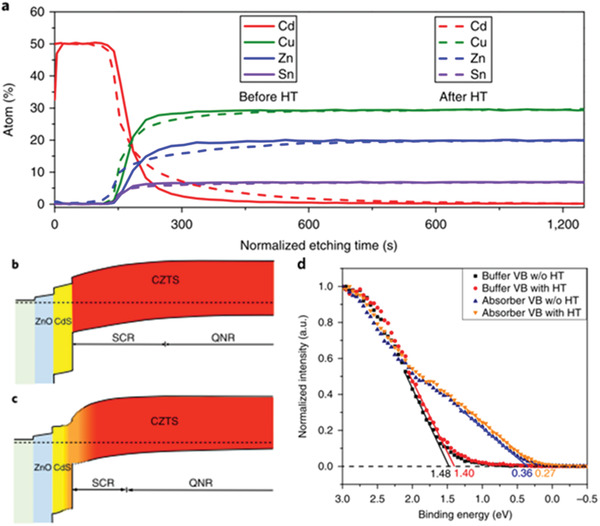
a) Elemental depth profiles of heterojunction with and without the HT process detected by XPS. b,c) Schema of conduction and valence band according to XPS measurements with and without the HT process. d) XPS valence band characteristics for CdS and CZTS thin film with and without HT. The solid lines show the linear extrapolation lines for XPS data close to the valence band maximum. Reproduced with permission.^[^ ^37^ ^]^ Copyright 2018, Springer Nature.

For the pure sulfide or sulfide‐rich kesterite, the conduction band offsets (CBO) of the heterojunction (kesterite/CdS) interface are cliff‐like, aggravating nonradiative recombinations and is detrimental to the *V*_oc_ and efficiency.^[^ ^35^ ^]^ There are generally two ways to re‐establish a favorable band alignment: i) applying alternative buffer materials with suitable conduction band energy such as Zn_1−_ *_x_*Cd*_x_*S, In_2_S_3_, and Zn_1−_ *_x_*Sn*_x_*O*_y_*;^[^ ^80^, ^81^, ^82^, ^83^ ^]^ ii) using HJT induced elemental interdiffusion to construct a continuous conduction band alignment.^[^ ^37^, ^39^, ^65^, ^66^, ^77^, ^78^, ^79^ ^]^ Tajima et al. first performed systematic research on the temperature‐dependent heterojunction treatment on CZTS solar cells.^[^ ^79^ ^]^ They confirmed that low temperature treatment at 200 °C could not lead to Cd diffusion into the absorber, whilst high temperature at 400 °C is harmful due to the segregation of ZnS secondary phases at the heterojunction interface after heat treatment.^[^ ^79^ ^]^ The accumulation of excess amounts of ZnS at the surface of kesterite is usually considered as a blocking barrier for electron transport.^[^ ^84^ ^]^ Therefore, a moderate treatment temperature around 300 °C is recommended to control the ZnS segregation whilst allowing sufficient Cd diffusion into the surface region of CZTS.^[^ ^79^ ^]^ In addition to the annealing temperature, the thickness of the CdS layer is also seen to be important, considering part of CdS will be consumed. Tajima et al. demonstrated a 9.4% efficient CZTS device by optimizing the HJT temperature and the thickness of CdS layer.^[^ ^77^ ^]^ A spike‐like CBO band alignment between CZTS and buffer layer was constructed due to the significant interdiffusion of Cd and Zn after HJT.^[^ ^77^ ^]^ This was also confirmed by the hybrid DFT calculation, where the conduction band minimum (CBM) of Zn_1−_ *_x_*Cd*_x_*S was found to move to a higher level with increasing Zn content.^[^ ^26^ ^]^ Our previous results experimentally confirmed that the Zn_1−_ *_x_*Cd*_x_*S buffer layer is able to tune the CBO with pure sulfide kesterite (CZTS) from cliff‐like to spike‐like.^[^ ^37^ ^]^ On the other hand, HJT can also facilitate the diffusion of Cd from the buffer layer into the surface of the kesterite absorber.^[^ ^37^ ^]^ The formation of a thin Cu_2_(Zn,Cd)SnS_4_ (CZCTS) layer within the top region of CZTS has been confirmed by several different characterization methods.^[^ ^37^, ^77^ ^]^ Despite the slightly decreased bandgap, the incorporation of Cd into CZTS has been proven effective to improve device performance by increasing the minority carrier lifetime and reducing band tailing.^[^ ^41^ ^]^ By carefully optimizing the HJT process, with controlled elemental interdiffusion and a favorable band alignment (**Figure** **3**), a CZTS solar cell with record champion efficiency of 11% has been demonstrated.^[^ ^37^ ^]^

Unlike the consistent benefits of HJT for the pure sulfide CZTS, the impact from the same treatment on selenide CZTSe/CdS devices appear to be different. The CBO of CZTSe/CdS is spike‐like according to both theoretical calculation and experimental observations, which is greatly different from the cliff‐like CBO of the sulfide kesterite solar cell.^[^ ^85^, ^86^, ^87^, ^88^ ^]^ Crovetto et al. reported that the boosted out‐diffusion of Zn from the Zn‐rich CZTSe absorber by HJT is highly likely to give an adverse impact due to the further increase of the CBO beyond the optimal range (0–0.3 eV).^[^ ^35^ ^]^ However, some literature reports enhanced performance of CZTSe solar cell resulting from heterojunction treatment.^[^ ^66^ ^]^ Neuschitzer et al. optimized the annealing temperature and duration of the treatment on CZTSe solar cell, with all performance parameters steadily increasing with annealing temperature up to 200 °C.^[^ ^66^ ^]^ This may be attributed to the reduced interface defects after an appropriate Cd and Cu interdiffusion and the out‐diffusion of Zn near to the surface.^[^ ^66^ ^]^

The HJT can be processed either in the air or inert gas. Giraldo et al. pointed out that the performance of a device treated in the air slightly outperformed that of Ar.^[^ ^89^ ^]^ To compare the potential difference resulting from the annealing atmosphere, here, we summarized experimental HJT conditions in **Table** **3**. It seems that sulfide kesterite prefers inert atmosphere, whereas selenide kesterite prefers to be annealed in air. The annealing temperature for sulfide kesterite seems to be higher than that for selenide. Such a difference might be attributed to the significant element interdiffusion required for the modification of CBO of sulfide CZTS/CdS devices. The cliff‐like CBO for sulfide kesterite will require a higher temperature to facilitate the elemental interdiffusion and the formation of new phases, such as (Zn,Cd)S and Cu_2_(Zn,Cd)SnS_4_ intermediate layers for re‐establishing a favorable band alignment. For CZTSSe, an intentional heterojunction heat treatment is rarely reported, though this treatment may unintentionally occur during the processing of the TCO window layer.

**Table 3:** Summary of heterojunction treatment on kesterite absorber/buffer layer classified by different atmospheres. The *V*_oc_ deficit is defined by Shockley–Queisser (S‐Q) limit

	Absorber	Optimized process conditions	Ref. efficiency [%]	Ref. *V*_oc_‐deficit	Efficiency [%]	*V*_oc_ [mV]	*V*_oc_‐deficit	*J*_sc_ [mA cm^−2^]	FF[%]	Ref.
N_2_ or Ar	CZTS	300 °C	/	/	7.0	610	/	19.7	58	^[^ ^79^ ^]^
	CZTS	330 °C for 20 min	5.0	/	9.4	700	531	21.3	63	^[^ ^77^ ^]^
	CZTS	270 °C for 10 min	7.82	558.5	11.01	730.6	500.4	21.74	69.27	^[^ ^37^ ^]^
Air	CZTSe	200 °C for 5 min	2.2	467	5.6	353	430	30.3	52.4	^[^ ^66^ ^]^
	CZTSSe	330 °C for 20 min	1.37	536	5.8	433	640	24.7	54.2	^[^ ^170^ ^]^

Heat treatment can also be applied to a completed device, which is referred to as device annealing. The device annealing process is also reported to give rise to pronounced improvement in device performance. The benefits arise from not only the modified bulk defects and heterojunction interface which are basically the same as HJT, but probably also the element interdiffusion at the buffer/TCO interface as well as the modified transparency and/or conductivity of the TCO window layer.^[^ ^65^, ^90^, ^91^, ^92^, ^93^, ^94^ ^]^ It is noteworthy that most reported successful device annealing cases are based on ITO window layer rather than an AZO. During the device annealing, the In element in the ITO window layer could diffuse into CdS buffer layer and subsequently increases the doping level of the CdS layer. This is expected to increase the built‐in electric field of the heterojunction, thus providing an additional benefit compared to those of postannealing and HJT. Indeed, the latest work from Su et al. demonstrated 12.6% Cd alloyed Cu_2_ZnSnS_4_ enabled by device annealing. They exclude i‐ZnO layer in device architecture to facilitate the interdiffusion of Cu, Zn, In and Sn, which improved the interfacial electron and hole densities. Consequently, a remarkable high FF of 71% and a low *V*_oc_ deficit (*E*_g_/*q* − *V*_oc_) of 700 mV have been obtained.^[^ ^94^ ^]^

All the above‐mentioned thermal treatments are effective when the defects and defect clusters with a high formation energy and/or undesirable band alignment are the dominating issues for the poor performance in kesterite, but such improvements could be very limited if the baseline performance is not subjected to these issues.

## 3 Alkali Treatments in Kesterite Solar Cells

**Figure 4:**
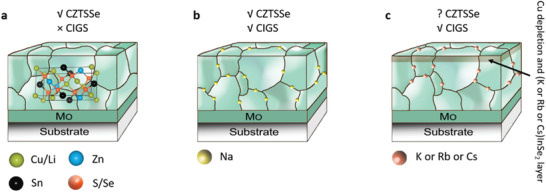
Schematics of alkali treatment for a) alloying Li and b) doping Na and c) K or Rb or Cs into CZTSSe and CIGS, respectively. The major benefit of Li is partial replacement of Cu to form Li alloyed CZTSSe. On the contrast, either alloying or doping Li in CIGS is detrimental to efficiency. The Na doping demonstrates positive impact both on CIGS and CZTSSe. Heavy alkali metals (K, Rb, and Cs) enable beyond 20% efficiency of CIGS solar cells. The studies of heavy alkali doping in CZTSSe show neglectable improvement.

The incorporation of alkali elements into the CIGS solar cells has led to two step‐changes: greatly improved bulk quality by Na doping developed in the 1990s, and surface passivation by heavy alkali postdeposition treatment (PDT) developed recently.^[^ ^9^, ^59^, ^95^, ^96^, ^97^, ^98^ ^]^ Na doping for CIGS has been proven effective in passivating charge compensating donor‐like defects such as In_Cu_ and Ga_Cu_, thus significantly improving the minority carrier lifetime and effective p‐type doping.^[^ ^99^, ^100^ ^]^ The heavy alkali based chalcopyrites such as KInSe_2_, RbInSe_2_, and CsInSe_2_ formed by PDT are effective epitaxial surface passivation layers, which also allow for a thinner CdS buffer layer and an associated reduction in photocurrent loss caused by parasitic absorption.^[^ ^101^, ^102^, ^103^, ^104^ ^]^ The heavy alkali PDT has led to the efficiency leap from ≈20% to beyond 23% in the past decade.^[^ ^9^, ^59^, ^97^, ^98^, ^104^, ^105^, ^106^, ^107^, ^108^ ^]^ Due to the structural similarities between kesterite and chalcopyrite, alkali treatments are expected to improve the performance of kesterite in a similar way, having been intensively researched. Schematic diagrams for summarizing the diffusion of alkali metals in kesterite and chalcopyrite solar cells are shown in **Figure** **4**.

**Figure 5:**
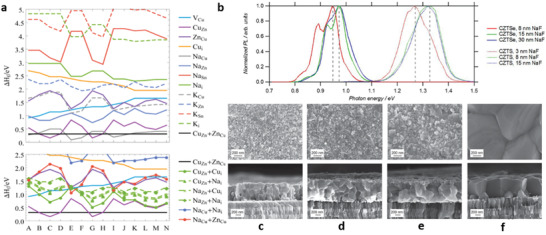
a) Calculated formation energy of (top) sodium and potassium associated point defects, and defect complexes (bottom) plotted at every extremum in the phase diagram, including related intrinsic defects for comparison. Reproduced with permission.^[^ ^109^ ^]^ Copyright 2018, AIP Publishing LLC. b) Normalized ambient temperature PL spectra of CZTSe and CZTS thin films with different initial Na contents. Reproduced with permission.^[^ ^117^ ^]^ Copyright 2015, AIP Publishing LLC. Top‐down (top) and cross‐sectional (bottom) SEM images of the sulfide CZTS thin films with different initial sodium floride contents: c) no NaF, d) 1 nm NaF, e) 4.5 nm NaF, and f) 23 nm NaF. Reproduced with permission.^[^ ^45^ ^]^ Copyright 2015, Wiley‐VCH.

The electronic defects introduced by Na and K in the kesterite system have been theoretically investigated by employing first‐principle calculations (**Figure** **5**a). The Na related antisite defects such as Na_Cu_, and Na_Zn_ have relatively low formation energies in the kesterite system so that their number could be large enough to change the electronic properties of the kesterite absorber.^[^ ^109^, ^110^ ^]^ The charge neutral Na_Cu_ antisites exhibit the lowest formation energy, which is even comparable with the most stable Cu_Zn_+Zn_Cu_ clusters, indicating Na can easily occupy Cu sites with large numbers. This Na‐Cu occupation may significantly suppress Cu‐absence induced lattice distortions and related disordering defects such as Zn_Cu_ and Sn_Cu_, thus effectively improving the carrier concentration.^[^ ^76^ ^]^ On the other hand, Na_Cu_ has much lower formation energy than V_Cu_ in the kesterite system, which means Na tends to remain at the Cu site after the quenching to room temperature, rather than moving out and enabling the formation of V_Cu_ which happens in chalcopyrite CIGS.^[^ ^111^, ^112^, ^113^ ^]^ The formation energy of acceptor Na_Zn_ antisite defects is higher than native acceptor Cu_Zn_ in kesterite.^[^ ^109^ ^]^ These theoretical results indicate the improvement of the p‐type doping density from Na doping in kesterite may not be as significant as that in CIGS. Due to the increased ion size, K‐related antisite defects such as K_Cu_ and K_Zn_ have much higher formation energies than those of Na‐related. This indicates that doping with K and heavier alkali elements seems unlikely to introduce a large number of extrinsic defects in the bulk of kesterite materials and is thereby unlikely to increase bulk minority carrier lifetime and p‐type doping density.

Experimentally, alkali doping for kesterite has also been intensively investigated.^[^ ^45^, ^114^, ^115^, ^116^ ^]^ Gershon et al. fabricated a highly Na‐containing CZTSe film by predepositing a NaF layer on Na‐free substrate. The introducing of NaF shows a strong influence on the electrical properties and morphology of CZTSSe absorbers (Figure 5b–f). A 30 nm NaF layer enabled a significant boost in *V*_oc_, fill factor (FF) and efficiency.^[^ ^117^ ^]^ Despite the unchanged carrier concentration with NaF, the improvement in performance could be attributed to the reduced nonradiative recombination, improved minority carrier diffusion length, and higher hole mobility.^[^ ^117^ ^]^ Some other groups also reported a long minority carrier lifetime in Na‐doped kesterite solar cells, with enlarged diffusion length and enhanced minority carrier collection efficiency.^[^ ^45^, ^117^, ^118^, ^119^, ^120^ ^]^ In terms of the effect of Na doping on grain growth and morphologies, controversial conclusions have been reported. Some groups reported the significant increase in grain size of kesterite by Na incorporation,^[^ ^45^, ^121^, ^122^, ^123^ ^]^ whilst some others reported no obvious change in grain size after Na incorporation.^[^ ^120^ ^]^ Gershon et al. have systematically investigated the amount of NaF on the morphology and related nonradiative recombination in kesterite. They found that a 4.5 nm or less NaF layer cannot significantly change the grain size.^[^ ^45^ ^]^ However, such a small amount of NaF layer is effective to suppress the nonradiative recombination, which was confirmed by temperature‐dependent PL spectra. A 23 nm NaF layer within the precursor can serve as a catalyst for crystal growth resulting in boosted grain size from several hundred nanometers to micrometers.^[^ ^45^ ^]^

K doping in solution‐based precursor or PDT is also extensively investigated.^[^ ^124^, ^125^, ^126^ ^]^ Similar to Na doping, K incorporation in the precursor with a suitably large amount is also proven to be effective in enhancing the grain growth of the kesterite absorber.^[^ ^125^ ^]^ Haass et al. reported that tenfold lower K could result in similar large grain size to that of Na for the solution‐based process.^[^ ^125^ ^]^ Though significant improvement of bulk minority carrier lifetime or p‐type doping by K doping in precursor has not yet been reported, PDT treatment with KF seems effective in reducing nonradiative recombination, as reported by Rey et al.^[^ ^127^ ^]^ However, the substrate temperature and atmosphere of their PDT process largely deviate from that typically performed for CIGS, and the baseline efficiency is relatively low. Besides, Haass et al. studied the similar Potassium PDT treatment on 10% efficient kesterite solar cell and proved the K‐PDT can readily reduce the interface recombination and boost *V*_oc_.^[^ ^128^ ^]^ However, the deposited KF layer with thickness >1 nm would lead to a current blocking phenomenon with decreased FF and current due to different surface chemistry compared to the CIGSSe case. Therefore, the whole process parameter or new K‐based PDT approach requires further optimization and development before realizing its full potential. Other heavy alkali dopants such as Rb and Cs have also been investigated by doping in solution‐processed precursors but have shown no improvements in device performance.^[^ ^46^, ^47^, ^116^ ^]^ These alkali elements (K, Rb, Cs) work well in CIGS only when introduced by PDT which, however, has not been intensively investigated for kesterite solar cells.

**Figure 6:**
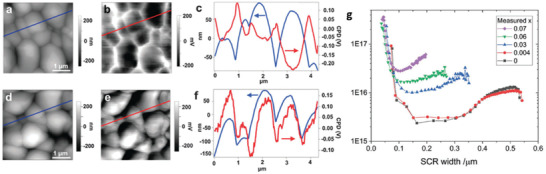
Thin film atomic force microscopy (AFM) topography images and SKPM potential maps. a) AFM topography, b) SKPM potential map, and c) plots of the topography and potential line scans of CZTSSe films without Li‐doping. d) AFM topography, e) potential map, and f) plots of the topography and potential line scans of CZTSSe films after Li‐doping. Reproduced with permission.^[^ ^45^ ^]^ Copyright 2015, The Royal Society of Chemistry. g) Apparent carrier concentration derived from room‐temperature C–V measurements for (Li*_x_*Cu_1−_ *_x_*)_2_ZnSn(S,Se)_4_ thin film solar cells. Reproduced with permission.^[^ ^48^ ^]^ Copyright 2018, Wiley‐VCH.

As the lightest alkali element, Li does not work in CIGS, but it does work in kesterite solar cells by either doping or alloying. As the radius of Li^+^ (0.90 Å) is very close to that of Cu^+^ (0.91 Å), Li atoms preferentially replace Cu on 2a sites thus can easily alloy into kesterite and form the (Li*_x_*Cu_1−_ *_x_*)_2_ZnSn(S,Se)_4_ compound in principle.^[^ ^48^, ^129^ ^]^ It has been experimentally confirmed that the bandgap of (Li*_x_*Cu_1−_ *_x_*)_2_ZnSn(S,Se)_4_ increases with the composition of Li by down‐shifting VBM.^[^ ^129^ ^]^ The bandgap of CZTS and Li_2_ZnSnS_4_ is 1.52 eV and 2.87 eV, respectively.^[^ ^130^ ^]^ Until now, several groups reported the highly efficient Li doped/alloyed kesterite solar cells.^[^ ^47^, ^48^, ^131^, ^132^, ^133^ ^]^ Xin et al. and Yang et al. found no significant bandgap change in CZTSSe in spite of different amounts of Li dopant added in the precursors, indicating that in some cases Li is difficult to be alloyed into the host lattice of CZTSSe.^[^ ^131^, ^134^ ^]^ This difficulty could be attributed to the Li‐Na ion exchange: the existence of heavy alkali Na stemming from glass substrates repels Li, similar to the K‐Na exchange in CIGS.^[^ ^58^ ^]^ By using SiO*_x_* as a Na and K diffusion barrier layer on Mo/SLG substrate, Li could be successfully alloyed into kesterite.^[^ ^129^ ^]^ Further investigation of the effect of small amounts of Li‐doping/alloying in CZTSSe shows that Li doping could effectively increase the p‐type doping of the absorber and lead to passivated grain boundaries showing upward band bending (**Figure** **6**c,f).^[^ ^131^ ^]^ This change of electrostatic potential at grain boundaries is not observed in Na doping. Despite the benefit of Li‐doping or alloying, Li‐loss during processing is a critical issue, which could account for up to 99% of Li loss from the initial precursor to the absorber.^[^ ^135^ ^]^ Li content in film continuously decreases at different fabrication stages. It is inevitable to lose Li during solution‐based processes, including solution‐based precursor coating and buffer layer deposition and the high temperature annealing process due to redissolution of Li salts and evaporation of the Li_2_Se phase, respectively.^[^ ^48^, ^131^ ^]^ In this regards, Zhang et al. developed a new Li doping method via selenization of the metal precursor by LiF and Se powder, which minimize the Li loss as the samples do not need to undergo an aqueous soaking process.^[^ ^133^ ^]^

## 4 Band Graded Kesterite Absorber via Substitution of Anion or Cation

Bandgap grading in the absorber layer is another key step‐change technology for high efficiency CIGS solar cells.^[^ ^136^, ^137^, ^138^, ^139^, ^140^, ^141^, ^142^, ^143^ ^]^ The notch like graded bandgap can boost *V*_oc_ by increasing the recombination energy barrier at the junction interface, whilst retaining low optical bandgap for large *J*_sc_, thus overcoming the trade‐off between *V*_oc_ and *J*_sc_ defined by the S‐Q limit of a single junction. The back grading of conduction band can act as a quasi‐electric field which could effectively facilitate the collection of minority carriers generated far away from the heterojunction region.^[^ ^138^, ^139^, ^140^, ^141^, ^144^, ^145^ ^]^ The front and back band gradings are enabled by the variations of composition depth profiles such as Ga/(In+Ga) and/or S/(Se+S).^[^ ^9^, ^136^, ^137^, ^140^, ^141^, ^142^, ^143^, ^146^ ^]^ This advanced strategy is also applicable to kesterite solar cells as the flexibility of anion and cation substitution could offer a variety of options for band grading.

The variation in anion composition (S/Se) enables the change of both CBM and VBM simultaneously, while the variation of cation composition changes either CBM or VBM.^[^ ^147^ ^]^ For instance, Ge substitution for Sn mainly changes the conduction band, and Ag substitution for Cu mainly changes the valence band.^[^ ^52^, ^53^, ^148^, ^149^, ^150^ ^]^ Since the downward VBM grading near the back contact will act as a barrier for hole collection and is thereby detrimental to device performance, the S/Se and Ag/Cu composition gradings are not favorable for back band grading, but appropriate and effective for front band grading. A beneficial back grading of the conduction band can be achieved by an upward gradient of Ge/Sn ratio.

**Figure 7:**
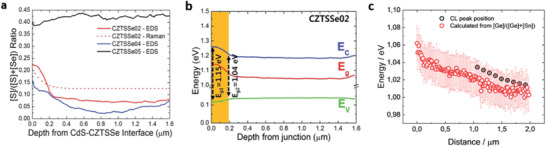
a) The [S]/([S] + [Se]) depth profile percentages of CZTSSe cells derived from the depth‐resolved Raman spectroscopy data. b) The band structure depth profile for 12.3% front grading CZTSSe solar cell. Reproduced with permission.^[^ ^151^ ^]^ Copyright 2016, The Royal Society of Chemistry. c) Cathodoluminescence (CL) peak position and bandgap value calculated from the Ge/Ge+Sn composition as a function of the distance from the back contact. Reproduced with permission.^[^ ^159^ ^]^ Copyright 2017, American Chemical Society.

The early efforts on the front band grading are based on surface S/Se grading by using H_2_S gas or other sulfur sources to realize a S‐rich surface.^[^ ^151^, ^152^, ^153^ ^]^ One challenge of this strategy is the control of graded diffusion depth. The optimal front band grading is a suitably steep grading within the depletion region, which is generally less than 300 nm for CZTSSe.^[^ ^144^ ^]^ However, the diffusion length of sulfur is usually too large if the sulfur source is introduced in the entire annealing process.^[^ ^151^ ^]^ On the other hand, the diffusion depth can be controlled by introducing the sulfur source after a high temperature annealing process. Cai et al. demonstrated a steep S‐rich surface in the CZTSSe absorber by introducing H_2_S gas during a cooling stage after selenization.^[^ ^152^ ^]^ DGIST used hybrid SeS_2_/Se as the chalcogenide source that offers another option for front grading, as the vapor pressure of Se is more sensitive to temperature than that of SeS_2_ and S.^[^ ^151^ ^]^ During the cooling stage of the annealing process, SeS_2_ and S dominate over Se as the main chalcogenide vapor, thus enabling a thin S‐rich surface (≈200 nm). The performance of CZTSSe solar cells was improved from 8.3% (ungraded) to 12.3% (graded) in their case through the fine control of the SeS_2_/Se ratio (in **Figure** **7**a,b), which clearly verified the benefit of S/Se front grading. Recently, front sulfur grading by surface sulfurization at room temperature using (NH_4_)_2_S as sulfur source is reported.^[^ ^154^, ^155^ ^]^ This approach enables a surface sulfurization without introducing additional defects by surface decomposition or Sn loss, whilst improves the band alignment of heterojunction interface, thus reducing interface recombination. Proper control of sulfurization duration and the amount of ammonia seems essential for optimizing this room temperature sulfurization process.^[^ ^154^, ^155^ ^]^

Ag/Cu grading is another promising option for front band grading, but also challenging due to the fast diffusion of both Cu and Ag during high temperature annealing. Using a solution‐based process, Qi et al. first reported successful front and back Ag graded (Cu_1−_ *_x_*Ag*_x_*)_2_ZnSn(S,Se)_4_ (ACZTSSe) solar cells.^[^ ^52^ ^]^ There are two key tricks that enable this Ag grading. First, the precursor is composed of multiple solution‐processed layers with different Ag contents in each layer. Moderate annealing (300–400 °C) on each of precursors formed an Ag contained crystallized compound to stabilize Ag atoms within the crystal structure. Second, a much lower annealing temperature of 480 °C (rather than the normal 550 °C) and an ultrafast ramping rate of 9 °C s^−1^ have been used to limit the interdiffusion of Ag and Cu.

**Figure 8:**
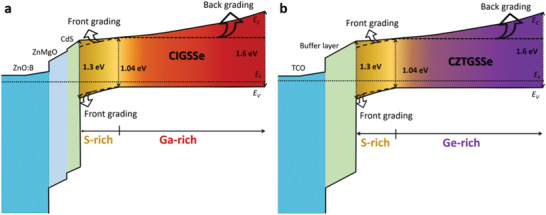
The double‐graded bandgap schematics of a) the state‐of‐the‐art CIGS thin film solar cell and of b) the proposed CZTGSSe thin film solar cells.

Considering the back band grading, Ge/Sn grading, which only changes the conduction band is an ideal option.^[^ ^156^ ^]^ By changing the Ge/Sn ratio, the bandgap of Cu_2_Zn(Sn,Ge)Se_4_ (CZTGSe) can be tuned from 1.07 to 1.44 eV, and that of Cu_2_Zn(Sn,Ge)S_4_ (CZTGS) can be tuned from 1.51 to 1.91 eV.^[^ ^150^ ^]^ The state of the art Ge homogeneous alloyed kesterite has achieved 12.3% efficiency with a fill factor of 72.7%, indicating that Ge alloying retains a high potential to produce a high‐quality absorber.^[^ ^157^ ^]^ Kim et al. demonstrated a back graded CZTGS solar cell based on nanocrystal‐based precursors. The bandgap of their absorber increases from 1.62 eV at the front side to 1.84 eV at the back.^[^ ^158^ ^]^ This graded bandgap effectively boosts both *J*_sc_ and *V*_oc_, leading to increased efficiency from 4.8% (ungraded reference) to 6.0%. The device performance of these cells is limited by the cliff‐like interface band alignment due to Ge alloying. IREC group reported that Ge naturally segregates toward the back of the absorber during annealing and thereby forms a back band grading (Figure 7c).^[^ ^159^ ^]^ Even though a Ge layer was intentionally stacked on top of the precursor, element interdiffusion during the selenization process still led to the segregation of Ge at the bottom. This segregation behavior of Ge is very similar to the segregation of Ga in CIGS.^[^ ^145^, ^160^, ^161^ ^]^ Therefore, a favorable conduction back band grading similar to that of high efficiency CIGS solar cells seems also achievable with Ge/Sn composition grading in CZTSSe. However, it should be noted that a significant amount of voids will form at the bottom in the heavily Ge‐alloyed kesterite due to the volatile Ge(S,Se)*_X_* phases.^[^ ^159^ ^]^ Modifying the growth process (e.g., introducing Ge(S,Se)x in chalcogenide atmosphere, reducing processing temperature,^[^ ^162^ ^]^ etc.) could be a promising approach to suppress the formation of voids when a large amount of Ge is alloyed. For front grading, using H_2_S as the sulfur source is a feasible method to realize a steep S/Se front grading. The Ge/Sn back grading and S/Se front grading in CZTSSe are very similar to the Ga/In back grading and S/Se front grading of the beyond 22% efficient CIGS solar cells fabricated by Solar Frontier (**Figure** **8**a,b).^[^ ^9^ ^]^ However, a notch‐like double bandgap grading is still a big challenge in CZTSSe. For instance, the fast replacing speed of Se for S makes it difficult to control the ideal S/Se grading depth profile; and the fast interdiffusion of Ag and Cu makes steep Ag/Cu grading very challenging. These strategies for bandgap grading are very promising but require a novel approach to fulfill their full potential.

## 5 Summary and Future Perspectives

In summary, this review presents the insights into the recent cutting‐edge strategies and research directions in the field of kesterite thin film solar cells, with particular focus on the postannealing treatment of absorber/heterojunction/device, alkali doping/alloying, and absorber energy band grading. Though, kesterite thin film solar cells have shown huge potential to be a cost‐effective, stable and environmental‐friendly thin film PV technology for terawatt level deployment, the existence of abundant detrimental bulk and interface defects and associated severe nonradiative recombinations hinder its photoelectric conversion property, featured with a large *V*_oc_ deficit, which has become the major challenge in this field.

Considerable efforts have been made to overcome this challenge. Postdeposition annealing treatments for bare absorber, heterojunction, or completed device have shown significant effectiveness in modifying the electronic quality of the kesterite absorber and interfaces with pronouncedly suppressed nonradiative recombination, leading to the record 11% efficiency of sulfide CZTS solar cells. The postdeposition thermal treatment on the absorber introduces the additional activation and relaxation of metastable defects and defect clusters which have relatively high formation energies, and thereby lead to the order‐disorder transition when a different quenching process is applied. This treatment is particularly effective for the alleviation of the metastable defects formed due to the insufficient relaxation during the material growth process. The thermal treatment of heterojunctions or completed devices not only offers the same mechanism for the relaxation of metastable defects as that on a bare absorber but also improves the heterojunction quality by facilitating the favorable element interdiffusion at the hetero‐interfaces. However, the thermal treatment itself cannot alleviate the defects and defect clusters with low formation energy, such as the thermal dynamically stable 2Cu_Zn_+Sn_Zn_ clusters that are responsible for the bandgap and/or electrostatic potential fluctuation. Therefore, this strategy seems unlikely to cope with the issue of potential fluctuation for the kesterite absorber.

Based on the current understandings and perspectives, gaining control of the intrinsic defect and defect cluster is a serious challenge in developing high‐performance kesterite solar cells. Experimental studies identify the formation of intrinsic point defects near the front interface (e.g., Fermi level pinning due to Cu‐Zn antisite defects) and within bulk (bulk recombination due to deep defects) as the key culprits behind the undesirable performance of solar cells. In order to suppress these defects, understanding its formation mechanism during the synthesis of kesterite phase is essential to the ultimate optimization for the fabrication process. The current two‐step (precursor deposition/sulfo‐selenization) fabrication processes facilitate the formation of deep defects, leading to a highly defective kesterite structure. Recently, Li et al. reported a new strategy to suppress the formation of the detrimental 2Cu_Zn_+Sn_Zn_ clusters and associated potential fluctuation,^[^ ^163^ ^]^ which is realized by engineering an appropriate local chemical environment of Zn‐rich and Cu‐poor with Sn oxidized to Sn^4+^ at the point in time when the synthesis of CZTSe phase initiates. This work provides a new promising direction to control the formation of detrimental intrinsic defects – engineering the local chemical environment during the growth of kesterite materials. Thus, developing/optimizing the absorber synthesis process still offers a fundamental and prospective pathway to realize high performance. Moreover, the surface passivation is an alternative way to alleviate the defects, particularly at the front interface. Postdoping an additional passivator (e.g., alkali elements),^[^ ^128^ ^]^ applying a passivation layer (e.g., Al_2_O_3_)^[^ ^164^ ^]^ and post‐treatment (oxygen plasma treatment)^[^ ^165^ ^]^ have been suggested in recent reports, demonstrating pronounced improvements in electronic properties and device performance. Therefore, integrating the bulk defects control with additional passivation steps will be crucial for delivering a step‐change in kesterite's performance.

Alkali doping is expected to passivate the bulk and surface defects of the kesterite absorber as it does in CIGS due to the great structural similarity of these two kinds of materials. Na and Li dopants have shown their effectiveness in reducing nonradiative recombination centers and enhancing the minority carrier lifetime and p‐type carrier doping density, leading to CZTSSe solar cells with beyond 12% efficiency. However, the heavy alkali dopants such as K, Rb, and Cs seem not to be as effective as Na and Li by doping in precursors. Nevertheless, the potential of these heavy alkali dopants for surface passivation using PDT treatment similar to that of CIGS has not been sufficiently exploited. The key to passivating the detrimental surface defects of kesterite using heavy alkali elements is to establish a suitable doping strategy, which enables the formation of alkali‐involved chalcogenide phases similar to the formation of KInSe_2_, RbInSe_2_ and CsInSe_2_ nanolayers during the surface PDT.

Constructing a notch‐like bandgap grading for kesterite absorber is another promising strategy to overcome the large *V*_oc_ loss of kesterite solar cells. The isovalent cation and anion mutations of kesterite offer great flexibility for bandgap grading. As postselenization or ‐sulfurization is a necessary step for high performance devices, the critical challenge of bandgap grading is to finely control the diffusion of alloying elements during the high‐temperature annealing. S/Se alloying for front bandgap grading has been successfully demonstrated. Ge/Sn alloying for back grading can form naturally, very similar to the Ga/In back grading in CIGS. These results indicate that by Ge/Sn back grading and postsurface sulfurization (or sulfurization after selenization, SAS), the strategy for notch‐like bandgap grading successfully demonstrated by Solar Frontier (Ga/In back grading and postsurface sulfurization) can also be applied to kesterite solar cells and is highly promising for realizing a high efficiency kesterite solar cells.

To move forward, the formation mechanisms of the detrimental defects and defect clusters need to be further investigated, so that more effective strategies could be developed, to either eliminate these defects and defect clusters during the growth process or passivate these defects after deposition. Therefore, a more detailed understanding of the overall defect characteristics and their synergistic influence on device performance is critical and urgent, whilst more effective approaches to reduce the detrimental native defects and suppress the nonradiative recombination and bandgap/potential fluctuation are still waiting for more intensive investigation.

## Conflict of Interest

The authors declare no conflict of interest.
